# Determinants of serum aflatoxin B1 exposure among pregnant women in coastal Kenya: a cross-sectional study in Kwale County

**DOI:** 10.1186/s41182-026-01061-y

**Published:** 2026-08-21

**Authors:** Richard Wodah-Seme, Gibson Omwansa Javes, Juma Changoma, Betty Muriithi, Samya Rashid, Miho Sato, Kazuchiyo Miyamichi, Mami Hitachi, Wataru Kagaya, Tomonori Hoshi, Christine Bii, Violet Wanjihia, Satoshi Kaneko

**Affiliations:** 1 Graduate School of Biomedical Sciences, Nagasaki University, Nagasaki, Japan; 2 Department of Eco-Epidemiology, Institute of Tropical Medicine, Nagasaki University, Nagasaki, Japan; 3 NUITM-KEMRI Project, Institute of Tropical Medicine (NUITM), Nagasaki University, Nairobi, Kenya; 4 School of Tropical Medicine and Global Health, Nagasaki University, Nagasaki, Japan; 5 Centre for Microbiology Research, Kenya Medical Research Institute (KEMRI), Nairobi, Kenya; 6 Centre for Public Health Research, Kenya Medical Research Institute (KEMRI), Nairobi, Kenya

**Keywords:** Aflatoxin B1, Serum biomarkers, Pregnant women, Geographical variation, Awareness, Public health, Kwale, Kenya

## Abstract

**Background:**

Aflatoxin B1 (AFB1) exposure is a major environmental health concern among vulnerable groups such as pregnant women, yet its determinants in coastal settings remain poorly understood. We aimed to quantify AFB1 exposure and examine its environmental, behavioral, and sociodemographic determinants among pregnant women in Kwale County, a predominantly rural area on Kenya’s southern Indian Ocean coast.

**Methods:**

In a facility-based cross-sectional study, serum AFB1–albumin adduct concentrations were measured by enzyme-linked immunosorbent assay in 298 pregnant women. Determinants were examined with a pre-specified multivariable linear regression of log-transformed concentrations using heteroscedasticity-consistent (HC3) standard errors, and a secondary Firth penalized logistic model of detectable versus non-detectable exposure.

**Results:**

Adducts were detectable in 251 participants (84.2%); the median concentration was 28.1 pg/mL (IQR 13.7–68.7). Exposure was determined primarily by where women lived: it was lowest in the community closest to the coast and progressively higher in two drier, more inland communities (2.02-fold, 95% CI 1.44–2.85; and 3.02-fold, 95% CI 1.89–4.82). Single marital status was associated with modestly higher exposure (1.89-fold; 95% CI 1.17–3.05). Food source, flour storage, maternal education, and age did not predict exposure, and the penalized logistic model reproduced the same geographical pattern. Despite near-universal exposure, only 1.7% of participants had ever heard of aflatoxin.

**Conclusions:**

AFB1 exposure was widespread and geographically structured, pointing to place-based rather than individual-level drivers and supporting area-targeted food-safety interventions to protect maternal and fetal health. These findings have implications for food-safety strategies in other tropical regions where climate and subsistence agriculture contribute to chronic aflatoxin exposure.

**Supplementary Information:**

The online version contains supplementary material available at 10.1186/s41182-026-01061-y.

## Background

Aflatoxins are naturally occurring mycotoxins produced primarily by *Aspergillus* species, particularly *Aspergillus flavus* and *A. parasiticus*, that commonly contaminate staple foods such as maize, groundnuts, and cereals in tropical and subtropical regions [1–3]. Among the aflatoxin subtypes, aflatoxin B1 (AFB1) is the most toxic and most extensively studied owing to its hepatotoxic, immunosuppressive, and carcinogenic effects [4, 5]. Human exposure occurs mainly through consumption of contaminated foods and indirectly through animal-source products derived from livestock fed contaminated feed [6, 7].

Environmental and post-harvest conditions strongly shape contamination. Temperature, humidity, drought stress, and poor storage promote fungal growth and toxin production, while inadequate post-harvest handling and weak food-safety systems further increase exposure risk in low- and middle-income countries [8–10]. Climate change is expected to intensify contamination risk, particularly across maize-dependent regions of sub-Saharan Africa where environmental conditions and agricultural systems favor fungal proliferation [11, 12].

After the consumption of contaminated food, AFB1 is rapidly absorbed from the intestines and transported to the liver, where it undergoes biotransformation by cytochrome P450 enzymes (CYP1A2 and CYP3A4) into reactive and non-reactive parts. The CYP3A4-mediated reactive component AFB1-8,9-epoxide (AFBO) forms covalent adducts with cellular macromolecules, including DNA and proteins. While some metabolites are detoxified through glutathione conjugation and subsequently excreted in urine or bile, a proportion of the reactive epoxide binds irreversibly to serum albumin, forming aflatoxin B1–albumin adducts. Because human serum albumin has a biological half-life of approximately 20 days, these adducts remain detectable for approximately 2–3 months and provide an integrated measure of recent chronic exposure rather than reflecting only recent dietary intake [13–15].

In addition to epidemiological evidence, aflatoxin B1 is supported by extensive toxicological data demonstrating its potent genotoxic and carcinogenic properties. Experimental studies in multiple animal models show that AFB1 induces dose-dependent hepatocellular carcinoma, driven by the formation of AFB1-N7-guanine DNA adducts and the characteristic G→T transversion at codon 249 of the *TP53* gene (the “serine mutation”), a hallmark lesion also observed in human liver tumors [16]. Because AFB1 acts through a non-threshold genotoxic mechanism, international risk-assessment bodies—including JECFA and WHO—have concluded that no Acceptable Daily Intake (ADI) or Provisional Tolerable Weekly Intake (PTWI) can be established for aflatoxins [6]. Instead, aflatoxin risk assessment relies on benchmark dose modeling, cancer potency factors, and margin-of-exposure approaches [17]. These toxicological findings provide strong causal support for epidemiological associations between aflatoxin exposure and adverse health outcomes.

Pregnant women are especially vulnerable because aflatoxins may adversely affect both maternal and fetal health. Chronic gestational exposure has been associated with immunosuppression, micronutrient deficiencies, intrauterine growth restriction, and low birth weight, and aflatoxin metabolites can cross the placental barrier and may be transmitted during breastfeeding, extending exposure during critical developmental periods [18–20].

Kenya remains among the countries most affected by aflatoxin contamination, with recurrent outbreaks of acute aflatoxicosis and evidence of widespread chronic exposure [21, 22]. This geographic concentration of outbreaks has led to disproportionate surveillance, intervention programs, and epidemiological research in these regions to the neglect of coastal counties such as Kwale [23]. Yet Kwale County, on Kenya’s southern Indian Ocean coast, may face elevated risk because persistent humidity, high temperatures, and common household storage practices create favorable conditions for fungal proliferation.

Recent studies have confirmed aflatoxin contamination across multiple components of Kwale’s food system: staple cereals consumed by households, aflatoxigenic *Aspergillus* species within the cashew nut value chain, and aflatoxin M1 in raw cattle milk alongside elevated aflatoxin B1 concentrations in dairy feed concentrates [24–27]. These findings suggest that residents may be exposed through several dietary pathways. Despite this emerging evidence, biomarker-based studies characterizing environmental, behavioral, and socioeconomic determinants of internal exposure among pregnant women in coastal Kenya remain limited. Existing research has largely focused on food contamination or geographically restricted populations without linking measured internal exposure to its determinants [18, 19]. Biomarker approaches—particularly serum AFB1–albumin adducts—integrate exposure from multiple dietary sources and account for inter-individual differences in absorption, metabolism, and detoxification. Numerous studies have demonstrated strong correlations between serum albumin adduct concentrations and chronic dietary exposure, making them more informative than food-contamination measurements alone. Although immunoassays do not distinguish all structurally related adducts, they are well validated for population-based exposure assessment and remain practical tools for evaluating chronic aflatoxin exposure in field settings [17, 20, 21].

Despite growing evidence of contamination within Kwale’s food system, no published studies have quantified serum AFB1–albumin adduct concentrations among pregnant women or examined determinants of exposure in this vulnerable population. Addressing this gap is essential for informing targeted public-health interventions, strengthening food-safety policies, and reducing the long-term health consequences of chronic aflatoxin exposure in coastal Kenya.

We therefore aimed to characterize serum AFB1 exposure and its determinants among pregnant women in Kwale County, on the southern coast of Kenya. Specifically, we aimed to (i) quantify internal AFB1 exposure using serum AFB1–albumin adducts; (ii) identify its environmental, behavioral, and sociodemographic determinants, distinguishing individual-level practices from the broader geographical and environmental context; and (iii) assess local awareness of aflatoxin, thereby informing area-based public-health and food-safety policy to protect maternal and fetal health in this and comparable tropical settings. In doing so, we sought to inform targeted, place-based public-health interventions to protect maternal and fetal health.

## Methods

**Fig. 1:**
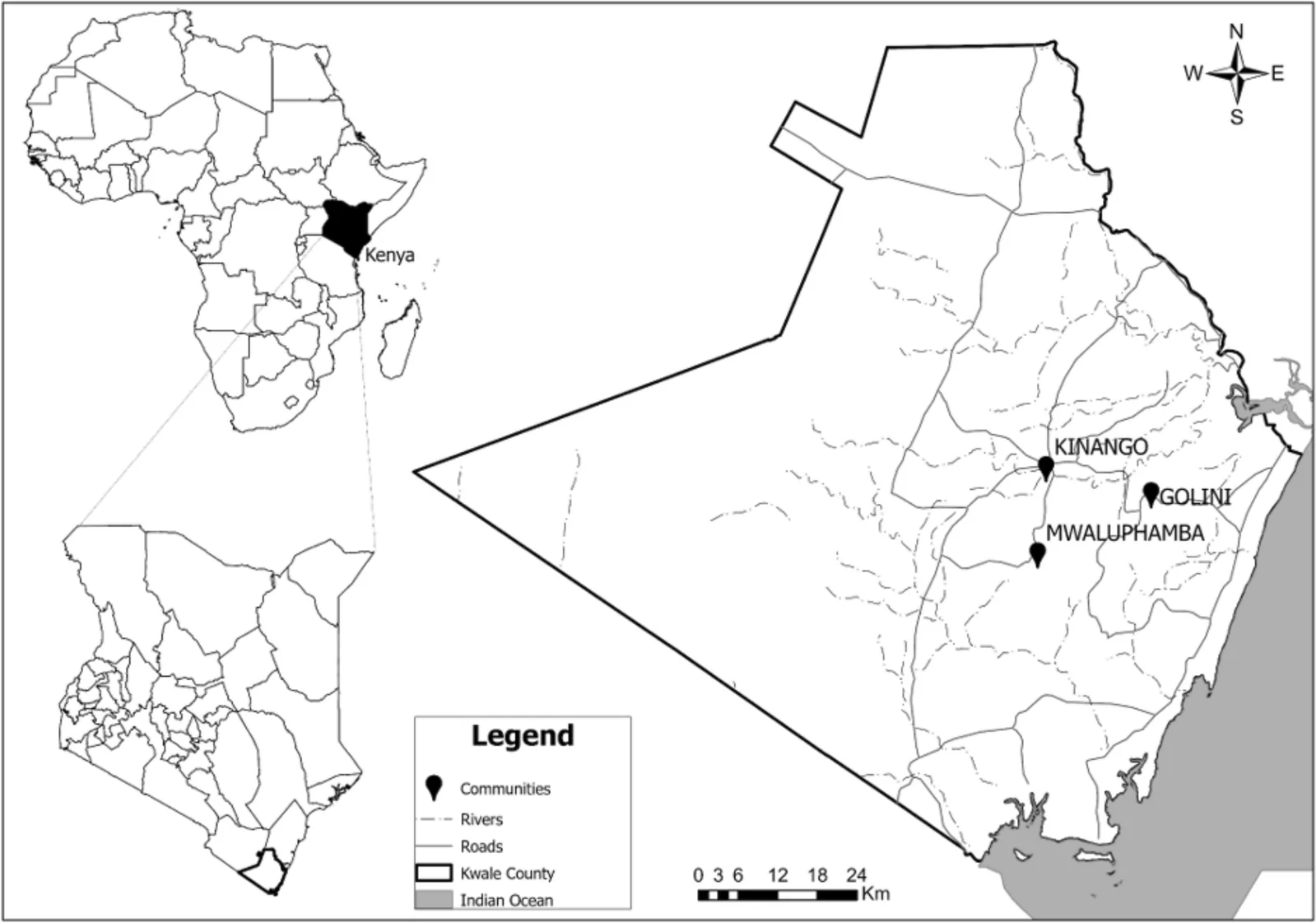
Location of the study sites. The panels show the three study communities—Golini, Kinango, and Mwaluphamba—in relation to other African countries (**A**) and within the administrative (county) boundaries of the study area (**B**). The sites lie within the Health and Demographic Surveillance System (HDSS) operated jointly by the Nagasaki University Institute of Tropical Medicine (NUITM) and the Kenya Medical Research Institute (KEMRI). The map was produced in ArcGIS Pro (Esri) using administrative boundary basemaps obtained from DIVA-GIS (https://www.diva-gis.org), which distributes GADM data under the terms described at https://gadm.org/license.html

This was a facility-based cross-sectional study conducted among pregnant women attending antenatal care clinics in Kwale County, on the southern Indian Ocean coast of Kenya. The study was conducted within a cohort area of the Nagasaki University Institute of Tropical Medicine and the Kenya Medical Research Institute Health and Demographic Surveillance System (HDSS), covering approximately 384.9 km^2^ across three communities, Golini, Mwaluphamba, and Kinango, within Kwale County, a predominantly rural area south of the port city of Mombasa, from September 2023 to May 2024 (Fig. 1) [28]. The three communities lie along a short coastal-to-inland gradient that is central to the patterns reported here: Golini, nearest the coast, sits beside the forested and humid Shimba Hills, whereas Kinango and Mwaluphamba lie further inland in drier, more open and deforested terrain.

Kwale County faces the Indian Ocean to the east and southeast, and Tanzania to the southwest. The county has a tropical climate characterized by bimodal rainfall, with long rains from March to June and short rains from October to December. Annual rainfall ranges from 900 to 1500 mm, with average temperatures between 24 °C and 30 °C and consistently high humidity [29]. These conditions favor fungal proliferation, including Aspergillus species known to produce aflatoxins [30]. The study site lies within Kenya’s aflatoxin-prone belt, where climatic conditions and reliance on susceptible staple crops converge to increase population exposure.

Agriculture is central to livelihoods in the county. Major staple crops include maize, cassava, cowpeas, and groundnuts. Most households rely on subsistence farming, producing, storing, and consuming their own food. Post-harvest practices such as sun-drying on open surfaces and storage under suboptimal conditions increase the risk of fungal contamination and aflatoxin production. Limited access to improved storage technologies and rainfall variability further exacerbate this risk [31, 32].

In 2017, Nagasaki University, through its collaboration with the Kenya Medical Research Institute, established a cloud-based electronic data-capture system, the Pregnant Women and Infant Registration (WIRE) system. The system assigns a unique identifier to each pregnant woman and tracks her from pregnancy to delivery; at delivery, the infant is also assigned a unique identifier used throughout the postnatal period until the fifth birthday [33]. This system facilitates longitudinal follow-up and data integration, providing a robust platform for population-based research.

We leveraged the WIRE system to recruit pregnant women attending antenatal care (ANC) services at three primary health facilities within the surveillance area. Eligible participants were in their second or third trimester, 18 years of age or older, not carrying multiple pregnancies, not on any ongoing nutrition intervention, and registered in the WIRE system. The system provided baseline demographic and clinical information, enabling efficient participant identification within an ongoing prospective birth cohort.

Trained research assistants collected additional data not captured in the WIRE system through face-to-face interviews during scheduled ANC visits, including the methods used to identify spoiled maize and how such maize was handled. Venous blood (5 mL) was collected by trained phlebotomists using sterile technique into red-top tubes (BD 367814) with clot activator. Samples were allowed to clot at room temperature for 30–60 min and then centrifuged at 3500 rpm for 10 min to separate serum. Serum was aliquoted into labeled vials using sterile pipette tips and stored at − 40 °C. Samples were transported on dry ice to Kenya Medical Research Institute laboratories in Nairobi for storage and analysis; laboratory analyses were conducted between February and May 2024.

Serum AFB1–albumin adducts were selected as the biomarker of exposure because they provide an integrated measure of chronic internal aflatoxin exposure over approximately the preceding 2–3 months, reflecting the approximately 20-day biological half-life of human serum albumin [15]. Following hepatic metabolism of aflatoxin B1, the reactive AFB1-8,9-epoxide forms stable covalent adducts with serum albumin, making these adducts suitable for assessing cumulative exposure in epidemiological studies [14]. Compared with urinary biomarkers, such as aflatoxin M1 or AFB1–N7-guanine, which primarily reflect recent exposure within the previous 24–48 hours, serum albumin adducts are less affected by day-to-day dietary variation and therefore provide a more reliable estimate of habitual exposure [34]. Serum AFB1–albumin adducts have been extensively validated and are widely used in population-based studies investigating chronic aflatoxin exposure.

Serum AFB1–albumin adduct concentrations were quantified using a commercial ELISA kit (GloryScience Co., Ltd.) following the manufacturer’s protocol with minor modifications [28]. The assay reflects exposure over the preceding 2–3 months. The limit of detection (LOD) was calculated as LOD = 3.3 × (standard deviation of the intercept)/(slope of the standard curve); the calculated LOD was 8 pg/mL. Left-censored values were imputed as half the LOD (4 pg/mL) for descriptive summaries.

All analyses were performed in R version 4.4.1. Continuous variables were summarized as medians with interquartile ranges (IQR) and categorical variables as counts and percentages. The proportion of samples with detectable adducts (≥ LOD) was reported overall and for each community.

Distribution and transformation. Serum AFB1–albumin adduct concentrations were strongly right-skewed and were therefore analyzed on the log10 scale. Concentrations below the LOD (8 pg/mL; 47/298, 15.8%) were imputed at half the LOD (4 pg/mL) for descriptive summaries and for the primary model, with alternative substitutions examined in sensitivity analyses.

The primary analysis was a multivariable linear regression of log10 AFB1 on a set of covariates specified a priori to represent environmental and behavioral exposure domains: geographical location (Golini, Kinango, Mwaluphamba), source of foodstuff (farm, shop), flour-storage method (khaki bags, lidded plastic tins), maternal education (no formal education, any formal education), marital status (married, single), and maternal age group (18–24, 25–34, 35–50 years). Covariates were fixed in advance rather than chosen by data-driven (stepwise/AIC) procedures, which bias coefficients, produce over-optimistic confidence intervals, and yield unstable models when categories are sparse. Heteroscedasticity-consistent (HC3) standard errors were used. Because the residual variance was unlikely to be constant across communities of unequal size, statistical inference was based on heteroscedasticity-consistent (HC3) robust standard errors, which remain valid under non-constant error variance and are recommended for small-to-moderate samples. Regression coefficients (*β*) were exponentiated and are reported as fold-changes in AFB1 concentration (10^β^) that is, ratios of the geometric-mean concentration relative to the reference category, with 95% confidence intervals. Model adequacy was assessed using residual diagnostics, multicollinearity was checked with variance inflation factors, and the influence of the single most extreme observation was evaluated in sensitivity analyses.

Two secondary analyses were pre-specified. First, to model any versus no measurable exposure, a Firth penalized logistic regression contrasting detectable with non-detectable participants was fitted using the same covariates; Firth’s penalization was applied because the non-detectable group was small and several covariate strata were sparse, which produces separation (non-finite estimates) in standard maximum-likelihood logistic regression. Adjusted odds ratios are reported with profile penalized-likelihood 95% confidence intervals. Second, for descriptive purposes only, a Gaussian finite mixture model (mclust package) was fitted to the log10 concentrations of detectable samples, with the number of components selected by the Bayesian Information Criterion and below-LOD samples assigned to the lowest stratum; because the number and placement of components were unstable, the selected solution over-segmented an essentially continuous distribution, this classification was used only to describe exposure and not for inference.

Maternal age was missing for 15 participants (5.0%); all other variables, including maternal education, were complete. Maternal education was recorded on a four-level scale (no formal education, primary, secondary, tertiary); in the regression models it was entered as a binary contrast of any formal education versus no formal education. The primary model used complete cases for age (*n* = 283).

Robustness was assessed by (i) varying the substitution value for left-censored observations (LOD and LOD/√2), (ii) excluding the single extreme observation, and (iii) modeling maternal education under alternative classifications (three levels: no formal education, primary, and secondary or higher, and a binary contrast of secondary-or-higher versus primary-or-less). All tests were two-sided with significance set at *p* < 0.05. Analyses used the R packages mclust, logistf, sandwich, and lmtest.

## Results

### Participant characteristics and overall exposure

A total of 305 women were enrolled and 298 retained after exclusions (*n* = 7; incomplete serum samples and missing entries). Maternal age was missing for 15 participants (5.0%); other variables, including education, were complete. Serum AFB1–albumin adducts were detectable in 251 participants (84.2%); the median concentration was 28.1 pg/mL (IQR 13.7–68.7). Participant characteristics are summarized in Table 1.

**Table 1:** Socio-demographic, food-safety, and exposure characteristics of participants, by community

Characteristic	Golini (*n*=86)	Kinango (*n*=167)	Mwaluphamba (*n*=45)	Total (*n*=298)
Maternal age group (years)				
18–24	33 (38.4)	55 (32.9)	22 (48.9)	110 (36.9)
25–34	45 (52.3)	82 (49.1)	13 (28.9)	140 (47.0)
35–50	5 (5.8)	20 (12.0)	8 (17.8)	33 (11.1)
Missing	3 (3.5)	10 (6.0)	2 (4.4)	15 (5.0)
Marital status				
Married	78 (90.7)	155 (92.8)	41 (91.1)	274 (91.9)
Single	8 (9.3)	12 (7.2)	4 (8.9)	24 (8.1)
Maternal education				
No formal education	5 (5.8)	20 (12.0)	22 (48.9)	47 (15.8)
Primary	28 (32.6)	98 (58.7)	19 (42.2)	145 (48.7)
Secondary	33 (38.4)	32 (19.2)	3 (6.7)	68 (22.8)
Tertiary	20 (23.3)	17 (10.2)	1 (2.2)	38 (12.8)
Maternal employment				
Housewife	57 (66.3)	104 (62.3)	37 (82.2)	198 (66.4)
Working	29 (33.7)	63 (37.7)	8 (17.8)	100 (33.6)
Source of foodstuff				
Farm	39 (45.3)	133 (79.6)	44 (97.8)	216 (72.5)
Shop	47 (54.7)	34 (20.4)	1 (2.2)	82 (27.5)
Flour storage method				
Lidded plastic tins	52 (60.5)	167 (100.0)	41 (91.1)	260 (87.2)
Khaki bags	34 (39.5)	0 (0.0)	4 (8.9)	38 (12.8)
Handling of spoiled maize				
Fed to animals	6 (7.0)	131 (78.4)	10 (22.2)	147 (49.3)
Cannot identify spoiled maize	47 (54.7)	36 (21.6)	1 (2.2)	84 (28.2)
Disposed of it	30 (34.9)	0 (0.0)	20 (44.4)	50 (16.8)
Consumed it	3 (3.5)	0 (0.0)	14 (31.1)	17 (5.7)
Serum AFB1				
Detectable, *n* (%)	64 (74.4)	146 (87.4)	41 (91.1)	251 (84.2)
Median (IQR), pg/mL	21.2 (8.0–52.8)	36.6 (14.7–80.0)	51.3 (22.4–85.1)	28.1 (13.7–68.7)

^1^Values are n (% within community) unless otherwise stated; the Total column shows n (% of all participants)

In a 24-hour dietary recall, maize consumed as ugali was near-universal (292/298; 98.0%), whereas 58 (19.5%) reported eating nuts and 109 (36.6%) rice (Additional file 1: Table S1).

### Geographical variation in exposure

**Fig. 2:**
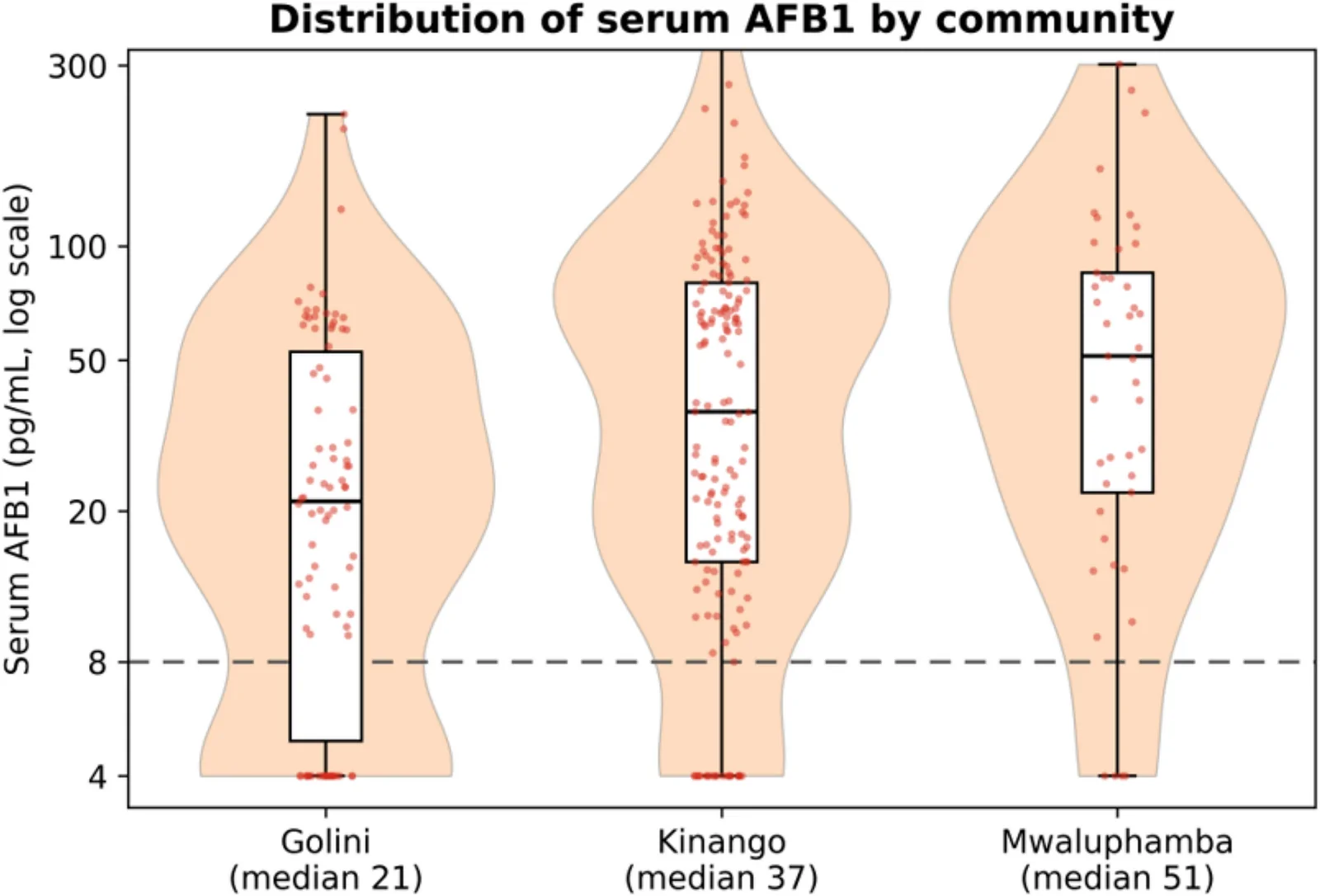
Distribution of serum AFB1–albumin adduct concentrations by community (log scale). Shaded areas are kernel density estimates; boxes show medians and interquartile ranges; points are individual participants; the dashed line marks the limit of detection (8 pg/mL)

Exposure differed markedly across the three communities, rising from the coastal community of Golini (median 21.2 pg/mL; 74.4% detectable) through the more inland community of Kinango (36.6 pg/mL; 87.4% detectable) to the more inland community of Mwaluphamba (51.3 pg/mL; 91.1% detectable) (Fig. 2).

### Determinants of exposure concentration

In the pre-specified multivariable model (Table 2), geographical location was the dominant determinant of exposure. Relative to the coastal community of Golini, AFB1 concentrations were 2.02-fold higher in the inland community of Kinango (95% CI 1.44–2.85; *p* < 0.001) and 3.02-fold higher in the inland community of Mwaluphamba (95% CI 1.89–4.82; *p* < 0.001). Single marital status was associated with modestly higher exposure (1.89-fold; 95% CI 1.17–3.05; *p* = 0.009). Source of foodstuff (shop vs. farm: 1.18-fold; 95% CI 0.80–1.73), flour-storage method, maternal education (any formal vs. no formal: 1.27-fold; 95% CI 0.77–2.10), and maternal age group were not significantly associated with exposure concentration.

**Table 2:** Determinants of serum AFB1 concentration (multivariable linear regression on the log10 scale; *n* = 283)

Predictor	Fold-change	95% CI	p
Geographical location			
Golini	1.00 (reference)	–	–
Kinango	2.02	1.44–2.85	<0.001
Mwaluphamba	3.02	1.89–4.82	<0.001
Source of foodstuff			
Farm	1.00 (reference)	–	–
Shop	1.18	0.80–1.73	0.402
Flour storage method			
Khaki bags	1.00 (reference)	–	–
Lidded plastic tins	0.80	0.47–1.39	0.433
Maternal education			
No formal education	1.00 (reference)	–	–
Any formal education	1.27	0.77–2.10	0.345
Marital status			
Married	1.00 (reference)	–	–
Single	1.89	1.17–3.05	0.009
Maternal age group (years)			
18–24	1.00 (reference)	–	–
25–34	1.23	0.92–1.64	0.169
35–50	1.23	0.69–2.20	0.476

Effects are fold-changes in concentration (ratio of geometric means, 10^β^) with Heteroscedasticity-consistent (HC3) robust 95% confidence intervals

Food source and flour storage showed no significant association with exposure. In addition to food source and household storage practices, we evaluated demographic characteristics, maternal education, marital status, household socioeconomic status, aflatoxin awareness, and geographic location as potential determinants of serum AFB1 exposure. While food source and storage variables were not significantly associated with detectable exposure in the adjusted analyses, geographic location remained the most consistent predictor of both detectable exposure and serum AFB1 concentrations. These findings suggest that factors operating beyond the household level may contribute more substantially to chronic aflatoxin exposure in this population.

This may partly reflect limited variability in storage practices and modest sample size, which could reduce statistical power to detect small effects.

### Determinants of detectable exposure

The same geographical pattern held in the secondary Firth model for detectable versus non-detectable exposure (Table 3): the odds of any detectable exposure were higher in the inland communities of Kinango (OR 3.13; 95% CI 1.45–6.74) and Mwaluphamba (OR 7.17; 95% CI 2.13–29.03) than in coastal Golini. Maternal education was not robustly associated with detectability. Although the odds of detectable exposure were nominally higher for women with any formal schooling than for those with none (OR 3.04; 95% CI 1.18–7.72), this estimate depended entirely on where the education boundary was drawn: it was null when secondary-or-higher was contrasted with primary-or-less (OR 0.98; 95% CI 0.48–2.06) and non-monotonic across three levels (carried mainly by women with primary schooling, OR 3.32; 95% CI 1.23–8.88). It also did not correspond to higher exposure concentration in the primary model. We therefore did not interpret education as a determinant of exposure (see Discussion). Food source, storage, marital status, and age were not significantly associated with detectability. Results were materially unchanged when the single extreme value (2167 pg/mL) was excluded. The descriptive mixture model did not yield stable, separable exposure strata and was therefore not used for inference.

**Table 3:** Determinants of detectable versus non-detectable exposure (Firth penalized logistic regression; *n* = 283)

Predictor	OR	95% CI	p
Geographical location			
Golini	1.00 (reference)	–	–
Kinango	3.13	1.45–6.74	0.004
Mwaluphamba	7.17	2.13–29.03	0.001
Source of foodstuff			
Farm	1.00 (reference)	–	–
Shop	1.15	0.47–3.12	0.773
Flour storage method			
Khaki bags	1.00 (reference)	–	–
Lidded plastic tins	0.60	0.17–2.02	0.412
Maternal educationᵃ			
No formal education	1.00 (reference)	–	–
Any formal education	3.04	1.18–7.72	0.022
Marital status			
Married	1.00 (reference)	–	–
Single	2.91	0.68–27.14	0.167
Maternal age group (years)			
18–24	1.00 (reference)	–	–
25–34	1.29	0.64–2.57	0.472
35–50	1.34	0.47–4.44	0.595

^a^Adjusted for but not interpreted: this estimate is highly sensitive to the education cut-point (null for maternal education (no formal, any formal), OR 0.98; non-monotonic across three levels) and is not regarded as a robust association (see Discussion)

### Awareness of aflatoxin

Awareness of aflatoxin was minimal. Although 214 women (71.8%) reported being able to recognize visibly spoiled maize, only 5 (1.7%) had ever heard of aflatoxin, and those same 5 were the only participants aware of any associated health effects—set against detectable internal exposure in 84.2% of the sample. Recognition of visible spoilage did not consistently translate into avoidance: 147 women (49.3%) reported feeding spoiled maize to animals and 50 (16.8%) discarding it, whereas 17 (5.7%) reported still consuming it, and the remaining 84 (28.2%) could not identify spoiled maize at all. Although visible spoilage does not reliably indicate aflatoxin contamination, it is widely used by households as a practical safety cue. We therefore assessed recognition and handling of spoiled maize to understand behavioral pathways that may influence exposure.

Awareness of aflatoxin itself was negligible across all three communities.

## Discussion

This study measured serum AFB1–albumin adducts, a validated biomarker of chronic aflatoxin exposure, among pregnant women in Kwale County. The results indicate that AFB1 exposure in this coastal population is widespread: adducts were detectable in the large majority of participants. This high detectable prevalence is consistent with the chronic, population-wide aflatoxin exposure repeatedly documented in Kenya and across sub-Saharan Africa and reinforces that internal exposure in coastal Kenya is the norm rather than the exception [21, 22, 35]. More importantly, the magnitude of exposure was structured principally by place of residence rather than by individual food-handling or sociodemographic characteristics, a pattern consistent across the primary continuous model and a secondary model of detectable versus non-detectable exposure. More recently, Osoro et al. measured serum AFB1–lysine adducts among pregnant women in Mombasa, on the same coast, in relation to birth outcomes [36]. Building on that work, ours is, to our knowledge, among the first studies to characterize internal AFB1 exposure with a serum biomarker and to weigh individual against place-based determinants among antenatal women in a predominantly rural coastal Kenyan setting. Detectable exposure was considerably more common in our population (84.2%) than in that Mombasa cohort (38.7%), although differences in biomarkers (albumin- versus lysine-adduct) and assays should be interpreted with caution, as the studies employed different analytical approaches. Whereas our study quantified albumin-bound AFB1 adducts using ELISA, Osoro et al. measured the AFB1–lysine adduct following enzymatic digestion of serum albumin using a different analytical platform. Although both biomarkers reflect chronic internal aflatoxin exposure, differences in assay methodology, analytical specificity, and calibration may contribute to differences in measured exposure levels; the contrast nonetheless points to substantial heterogeneity in exposure within the Kenyan coast. Previous Kenyan biomarker work has otherwise centered on eastern, aflatoxicosis-prone or clinical (liver-disease) populations and on national surveys, whereas coastal and antenatal populations have largely been assessed through food-contamination measurement rather than internal dose [22, 37].

Together, these findings indicate that chronic aflatoxin exposure among pregnant women in coastal Kenya remains substantial but is not geographically uniform. Rather, exposure appears to vary considerably across coastal settings, highlighting the importance of identifying local environmental and food-system factors that drive this variability.

Geographical location was the strongest determinant of exposure. Serum AFB1 concentrations were approximately two- and three-fold higher, respectively, in the two more inland communities (Kinango and Mwaluphamba) than in the community nearest the coast (Golini), and the same ordering was seen for the probability of any detectable exposure. Microclimatic factors such as rainfall, temperature, and humidity are well established to influence fungal ecology and toxin production across the food value chain [3, 10]. The three study locations differ markedly in this respect. Golini, near the Shimba Hills, has dense forest cover, abundant organic matter, cooler temperatures, and high humidity, whereas Kinango is more inland, with reduced vegetation, greater deforestation, and prolonged dry spells; Mwaluphamba is transitional between the two [29].

The pronounced geographical gradient observed in this study suggests that upstream environmental and agro-ecological factors may play a more important role in determining chronic aflatoxin exposure than household-level food sourcing or storage practices. Ecological differences among the study locations, including variations in temperature, rainfall, humidity, vegetation cover, and drought stress, are known to influence the growth of aflatoxigenic *Aspergillus* species and aflatoxin production during crop cultivation, drying, and storage [38, 39]. In addition, contamination may occur at multiple stages of the food value chain, including post-harvest handling, storage by farmers and traders, transportation, and food distribution, before commodities reach individual households [40, 41]. Consequently, women residing in Kinango and Mwaluphamba may experience greater chronic exposure despite reporting food sourcing and storage practices similar to those of participants in Golini. Consistent with this interpretation, household food sourcing and storage variables were not independently associated with serum AFB1 concentrations after adjustment for other covariates, whereas geographical location remained the strongest predictor of exposure. These findings suggest that household practices alone may be insufficient to explain individual exposure where contamination has already occurred earlier in the food production and distribution chain. This interpretation is further supported by previous studies from Kwale County demonstrating aflatoxin contamination across multiple components of the local food system, including staple cereals, the cashew nut value chain, dairy feed concentrates, and raw cattle milk, indicating that chronic exposure is influenced by place-based environmental conditions and broader food-system processes rather than household behaviors alone [26]. Nevertheless, the possibility that the study had limited statistical power to detect modest associations between certain household-level variables and serum AFB1 exposure cannot be excluded and should be considered when interpreting these findings.

Although Golini may provide environmental conditions that support fungal colonization, favorable conditions for fungal growth do not necessarily correspond to maximal aflatoxin production [42]. Climatic factors such as drought stress, elevated temperatures, and fluctuating moisture can influence both the abundance of aflatoxigenic *Aspergillus* species and the regulation of aflatoxin biosynthesis, with toxin production often increasing under environmental or host stress rather than under conditions optimal for fungal growth [43]. Because fungal abundance was not measured in the present study, we cannot distinguish whether the geographical differences in serum AFB1 concentrations reflect variation in the prevalence of aflatoxigenic fungi, differences in toxin production, or a combination of both.

The drier, drought-prone conditions of Kinango and Mwaluphamba may therefore promote toxin production, and warmer conditions there may also intensify the activity of crop-boring insects that damage grain and create entry points for fungal infection before and after harvest. Differences in agronomic responses to harsh conditions, such as premature harvesting and storage of inadequately dried grain, may further elevate contamination. Together, a plausible, place-based explanation for the geographical gradient in internal exposure.

The geographical gradient is unlikely to be purely climatic, however. The three communities also differ in settlement type and livelihood: Golini lies near Kwale town and Kinango is itself a local town center, whereas Mwaluphamba is a rural farming community, differences that imply systematic variation in income and in how households obtain and store food. This was evident in the data: food was sourced almost entirely from households’ own farms in rural Mwaluphamba (98%), compared with far greater reliance on shops in the more peri-urban Golini (55% shop-sourced). Greater dependence on home-grown maize plausibly drives the higher exposure observed in the rural community: where households rely on their own harvest, contamination is largely determined upstream, during post-harvest drying and on-farm grain storage, and subsistence production, lower incomes, and limited drying and storage capacity may converge to allow fungal growth and toxin accumulation before the grain is ever milled. Because food source was almost perfectly aligned with community, the two could not be statistically separated, so the geographical effect likely embodies these livelihood and food-system differences rather than agro-ecology alone. The individual and household socioeconomic indicators we could measure, maternal education, maternal employment, and spouse education and employment, were not associated with exposure after accounting for community; we nonetheless lacked a direct measure of household wealth or income. The geographical signal is therefore best interpreted as a composite of agro-ecological, socioeconomic, and food-system factors that cluster by community, rather than microclimate alone. Under any of these interpretations the dominant drivers are contextual rather than individual, which reinforces the case for area-based intervention.

Single marital status was associated with modestly higher exposure. This may reflect greater socioeconomic vulnerability and more constrained access to safe, good-quality food among unmarried women, although the cross-sectional design precludes causal inference and the estimate should be interpreted with caution.

By contrast, commonly assumed to influence exposure, source of foodstuff, household flour-storage method, and maternal age, were not associated with exposure concentration. The household flour-storage method we recorded, the final container in which milled flour is kept, was not associated with exposure; importantly, however, this captures only the last step in the chain and not the upstream post-harvest handling, drying, and grain storage at which aflatoxin contamination is largely determined, which we did not measure. Its null association therefore does not exclude post-harvest storage as a driver; rather, the decisive step was unobserved. Improved low-cost drying and storage technologies for home-grown grain remain plausible mitigation measures warranting dedicated evaluation [19, 44–46]. Maternal education was not robustly associated with exposure. It did not predict exposure concentration, and the one nominally significant contrast, higher odds of detectable exposure among women with any formal schooling than among those with none, proved entirely dependent on how the education categories were drawn. The same data yielded a significant association when any formal education was contrasted with none, no association when secondary-or-higher was contrasted with primary-or-less, and a non-monotonic pattern across three levels (driven mainly by the primary-schooling group). That a result flips between significant, null, and non-monotonic according to where a single category boundary is placed, together with the sparse non-detectable cells (roughly 10–20 women per education group), chance variation or residual confounding (for example, by diet, food sourcing, or socioeconomic circumstances correlated with schooling) rather than a genuine effect of education. We therefore do not interpret education as a determinant of exposure. Overall, exposure was governed more by where women lived than by their individual characteristics [47, 48].

A striking feature of this population was the mismatch between exposure and awareness. Detectable internal exposure was near-universal (84.2%), yet only 1.7% of women had ever heard of aflatoxin, and most relied on visible spoilage as their only cue to unsafe food, an unreliable signal, because aflatoxin contamination is frequently invisible, odorless, and tasteless. This disconnect between measured serum AFB1 levels and participants’ awareness underscores a central insight of the study: knowledge is an essential precursor to risk reduction, but awareness alone is insufficient [49]. Households often have limited control over contamination that occurs before food reaches them—during crop production, post-harvest handling, storage by farmers or traders, transportation, and retail marketing [15]. Economic constraints and restricted access to alternative food sources may further compel families to consume contaminated staples despite knowing the associated risks [47, 50]. These realities indicate that public health interventions must extend beyond educational campaigns. Effective strategies require improved post-harvest management, strengthened food-safety surveillance, routine monitoring of aflatoxin along the food value chain, and policies that expand access to safe food commodities.

Together with the finding that exposure tracked place of residence rather than individual knowledge or behavior, this suggests that awareness-raising or behavior-change messaging aimed at individuals is, on its own, unlikely to reduce exposure here; structural, area-level measures along the local food value chain are needed. The public-health significance is heightened because the affected population is pregnant women: even without clinical outcome data in this study, chronic gestational exposure at the levels observed is concerning given aflatoxin’s established associations with impaired fetal and child growth, low birth weight, and immune dysfunction [19, 35, 51].

These findings carry policy implications, particularly under climate change. Continued warming may heighten aflatoxin risk in vulnerable coastal regions such as Kwale, and Kenya’s history of aflatoxicosis outbreaks underscores the need for locally tailored, area-based prevention [12, 52–54]. Because exposure clustered geographically, interventions targeted at higher-burden, rural farming communities, in particular improving post-harvest drying and on-farm storage of home-grown maize, alongside food-safety surveillance, are likely to be more efficient than uniformly applied, individually focused messaging [55, 56]. Antenatal care, through which participants were reached, offers a practical platform both for exposure surveillance and for linking higher-risk communities to food-safety and post-harvest interventions; because the burden fell disproportionately on rural, subsistence-farming households, such targeting would also address an equity dimension of exposure.

This study directly measured serum AFB1 biomarkers rather than relying on dietary assessment, providing objective evidence of cumulative internal exposure among a population that is both vulnerable and under-represented in aflatoxin research [22, 57]. Analytically, exposure was modeled on its natural continuous scale using a pre-specified set of covariates and robust variance estimation, avoiding arbitrary exposure cut-points and data-driven variable selection; left-censored values and missing data were handled explicitly, and findings were stable in sensitivity analyses.

A major strength of the present study is the use of serum AFB1–albumin adducts as an objective biomarker of aflatoxin exposure. Unlike dietary questionnaires, which are subject to recall bias, seasonal variation, and inaccuracies in estimating aflatoxin intake from diverse food sources, serum AFB1–albumin adducts provide an integrated measure of the internal dose resulting from actual exposure. Similarly, measurements of aflatoxin contamination in food samples reflect contamination at a single point in time and may not accurately represent individual exposure because contamination is often heterogeneous within food batches, varies over time, and is influenced by food preparation, storage, and consumption patterns. In contrast, serum AFB1–albumin adducts are formed following hepatic metabolism of aflatoxin B1 and remain detectable for approximately 2–3 months due to the approximately 20-day half-life of human serum albumin. Consequently, they provide a reliable indicator of recent chronic exposure by integrating aflatoxin intake from multiple dietary sources while accounting for inter-individual differences in absorption, metabolism, and detoxification. These characteristics make serum AFB1–albumin adducts one of the most widely accepted biomarkers for epidemiological studies investigating chronic aflatoxin exposure.

Several limitations should be acknowledged. The cross-sectional design precludes causal inference. Exposure was assessed at a single time point with one biomarker. Upstream post-harvest practices, grain drying and on-farm storage of home-grown maize, where contamination is largely determined, were not directly characterized, nor were retail or vending practices. We also lacked a direct measure of household wealth or income; although the socioeconomic proxies available to us were not associated with exposure, we could not fully separate agro-ecological from socioeconomic contributions to the geographical gradient, and future work combining household geolocation with wealth indices and environmental covariates (such as rainfall, vegetation, and temperature) would help disentangle them. We also acknowledge the analytical limitation of the ELISA method. Despite providing a practical and validated approach for population-based exposure assessment, it may not be able to distinguish between structurally related aflatoxin–albumin adducts, including those derived from aflatoxin G1. Nevertheless, given the predominance of aflatoxin B1 in Kenyan food systems and the extensive validation of this biomarker in epidemiological studies, the assay remains appropriate for assessing chronic aflatoxin exposure.

Finally, although the sample size was adequate for the primary analyses, it may have limited statistical power to detect modest associations with some household-level factors, and the relatively small number of participants with non-detectable exposure resulted in wider confidence intervals. Potentially protective cultural practices were also not evaluated and warrant investigation in future qualitative studies.

## Conclusions

This study found that most pregnant women had detectable serum AFB1 concentrations, indicating widespread chronic aflatoxin exposure, with substantial geographical variation across the study area. Women residing in Kinango and Mwaluphamba had significantly higher exposure than those in Golini, whereas household food sourcing and storage practices were not independently associated with serum AFB1 concentrations after adjustment. Because exposure was structured primarily by place rather than by individual knowledge or behavior, and awareness was near-absent, individually focused education alone is unlikely to be sufficient. Reducing this preventable environmental risk to maternal and fetal health will require area-targeted structural measures—prioritizing improved post-harvest drying and on-farm grain storage in higher-burden rural communities—together with strengthened food-safety surveillance and integration of aflatoxin monitoring into antenatal and maternal–child health services. These findings are relevant to other maize-dependent, aflatoxin-prone settings across tropical sub-Saharan Africa, particularly as climate change intensifies contamination risk.

## Supplementary Information

Additional file 1.

## Data Availability

The data presented in this study are not publicly available because they contain personally identifiable information and were generated within an ongoing cohort study for which analyses are continuing. Deidentified data may be made available from the corresponding author on reasonable request, subject to the data-governance policies of the parent cohort and to approval by the relevant institutional review boards (the KEMRI Scientific and Ethics Review Unit and the Institute of Tropical Medicine, Nagasaki University).

## Abbreviations

AFB1: Aflatoxin B1
AIC: Akaike information criterion
ANC: Antenatal care
CI: Confidence interval
ELISA: Enzyme-linked immunosorbent assay
HC3: Heteroscedasticity-consistent (type-3) covariance estimator
HDSS: Health and Demographic Surveillance System
IQR: Interquartile range
KEMRI: Kenya Medical Research Institute
LOD: Limit of detection
NUITM: Nagasaki University Institute of Tropical Medicine
OR: Odds ratio
SERU: Scientific and Ethics Review Unit
WIRE: Pregnant Women and Infant Registration (system)
