# Supplementary material for: Determinants of serum aflatoxin B1 exposure among pregnant women in coastal Kenya: a cross-sectional study in Kwale County
Source: Trop Med Health. 2026 Aug 21;54:173. doi: 10.1186/s41182-026-01061-y (PMC13495407; doi:10.1186/s41182-026-01061-y)
Supplement: Supplementary file 1 [file 41182_2026_1061_MOESM1_ESM.docx]

**Additional file 1: Table S1.** Staple food consumption in the past 24 hours among participants, by community.

| Characteristic | Golini (n = 86) | Kinango (n = 167) | Mwaluphamba (n = 45) | Total (n = 298) |
| --- | --- | --- | --- | --- |
| Ate rice the previous day, yes | 15 (17.4) | 92 (55.1) | 2 (4.4) | 109 (36.6) |
| Ate nuts the previous day, yes | 13 (15.1) | 42 (25.1) | 3 (6.7) | 58 (19.5) |
| Ate ugali the previous day, yes | 82 (95.3) | 165 (98.8) | 45 (100) | 292 (98.0) |

Values are n (% within community); the Total column shows n (% of all participants). “Ugali” denotes the maize-based staple.
